# ICRPfinder: a fast pattern design algorithm for coding sequences and its application in finding potential restriction enzyme recognition sites

**DOI:** 10.1186/1471-2105-10-286

**Published:** 2009-09-11

**Authors:** Chao Li, Yuhua Li, Xiangmin Zhang, Phillip Stafford, Valentin Dinu

**Affiliations:** 1 Department of Biomedical Informatics, Ira A Fulton School Engineering, Arizona State University, Phoenix, AZ 85004, USA; 2 Center of Innovations in Medicine, Biodesign Institute, Arizona State University, Tempe, AZ, 85287, USA; 3 Center for Infectious Diseases and Vaccinology, Biodesign Institute, Arizona State University, Tempe, AZ, 85287, USA

## Abstract

**Background:**

Restriction enzymes can produce easily definable segments from DNA sequences by using a variety of cut patterns. There are, however, no software tools that can aid in gene building -- that is, modifying wild-type DNA sequences to express the same wild-type amino acid sequences but with enhanced codons, specific cut sites, unique post-translational modifications, and other engineered-in components for recombinant applications. A fast DNA pattern design algorithm, ICRPfinder, is provided in this paper and applied to find or create potential recognition sites in target coding sequences.

**Results:**

ICRPfinder is applied to find or create restriction enzyme recognition sites by introducing silent mutations. The algorithm is shown capable of mapping existing cut-sites but importantly it also can generate specified new unique cut-sites within a specified region that are guaranteed not to be present elsewhere in the DNA sequence.

**Conclusion:**

ICRPfinder is a powerful tool for finding or creating specific DNA patterns in a given target coding sequence. ICRPfinder finds or creates patterns, which can include restriction enzyme recognition sites, without changing the translated protein sequence. ICRPfinder is a browser-based JavaScript application and it can run on any platform, in on-line or off-line mode.

## Background

### Restriction enzymes in genetic engineering

Restriction enzymes and methylase are components of a bacterial mechanism aimed at resisting attack from bacteriophages and removing foreign viral DNA sequences. A restriction enzyme cuts a DNA molecule and forms a sticky or blunt end at each side of the incision site without damaging the nitrogenous bases. A DNA ligase can then splice a cut end to that of another DNA molecule. Each restriction endonuclease is an enzyme that recognizes a specific DNA sequence and cuts the DNA molecule at a particular position in relation to the recognition sequence, producing a blunt or overhanging end, depending upon the enzyme chosen. While restriction enzymes typically recognize specific short DNA sequences, the genetic code is redundant, with most amino acids being represented by more than one codon. Therefore, with creative use of sequence modifications, and use of synonymous codons, one can create restriction sites without changing the precise amino acid sequence coded for.

A DNA sequence can be synthesized by assembling short synthetic oligonucleotides using PCR amplification. Using this approach, several oligonucleotides with overlapping end sequences can be assembled into a whole DNA sequence [1]. DNA synthesis using this PCR-based ligation method, however, has some limitations. For example, it can only produce DNA sequences with length up to approximately 1.5 kbp. In long DNA segments, the primer extension will stop at an unpredictable position in the DNA sequence, and the probability of base pair mismatches will increase. Additionally, the proportion of GC content is limited to 40-60% by this approach. Due to such limitations, a long whole DNA can not be synthesized by PCR technology alone. Using restriction enzyme cutting technology, several DNA segments can be connected to form a longer full-length DNA sequence [2-4]. There is an increasing interest in artificially synthesizing in vitro large DNA constructs and even whole genomes with restriction enzyme cleavage followed by ligation of segments[2,5-12].

### Informatics support for restriction site analysis

There are several informatics tools for finding existing restriction enzyme recognition sites in a DNA sequence. NEBcutter works in browser-based client-server mode [13]. The server maintains a restriction enzyme database, and accepts a DNA sequence and several parameters from a user. After calculating, the server returns to the user the locations of recognition and cleavage sites and displays the sites in the user's browser. The NEBcutter algorithm for locating restriction sites is written using the gcc environment. Filtering and queue management tasks are also implemented in C. Graphical rendering of results is performed by the GD libraries. GD libraries allow functionality such as zoom in, zoom out, and automatic adjustment of the number of tags of recognized sites. The web user interface is dynamically generated by PHP scripts, and the web server is supported by Apache. NEBcutter supports manually input DNA sequences, a sequence loaded from a local file, or a sequence identifier. If a sequence identifier is used the server will retrieve data from NCBI databases. Linear and circular input sequences are both supported. The current version also supports a list of generally used genomes or plasmids as default input sequences.

NEBcutter is a powerful tool to locate restriction enzyme recognition sites. It supports a wide range of restriction enzymes. The illustration of results is flexible and clear. But NEBcutter has several limitations. The first is the potential for network bottlenecks. If the input sequence is large, such as that of a plasmid or a genome, the network response time becomes long and the process may even error out. The second limitation is that NEBcutter only locates recognition sites in the *original *DNA sequences as input. It does not support recoding to create a new restriction site. Sometimes users may need a specific restriction enzyme site at a certain location. NEBcutter provides no solution for such situations.

Another similar informatics toolkit, the Sequence Manipulation Suite (SMS), supports many operations for sequence analysis, including restriction site identification [14]. It is a JavaScript based Web application and runs on the client machine. Since there is no information transferring between server and client, it has no network delay. It supports both linear and circular sequence input. But, like NEBcutter, SMS only locates recognition sites in the original DNA sequences, with no support for designing alternative restriction sites created through silent mutations.

WatCut, created at the University of Waterloo, is another online tool for restriction enzyme analysis [15]. It supports functions both to locate restriction enzyme cleavage sites directly in a given DNA sequence and to search for potential restriction sites that can be created in a DNA sequence using silent mutations. Input DNA sequences can be typed in the online form or loaded from the local drive. Then six sequences, three in the forward strands and the other three in the reverse strands, are read with frame shifting. After one of the six sequences is chosen, candidate restriction sites are listed. Results can be displayed graphically or listed textually in a table. Although WatCut introduces support for silent mutations, the functionality is limited. It can only find restriction enzyme recognition sites in a DNA sequence of at most 100 nucleotides. Like NEBCutter, the tool needs a server with PHP support and runs in a client-server mode, with potential performance bottlenecks in server response and network bandwidth.

The increasing interest in artificially synthesizing in vitro large DNA constructs and even whole genomes with the aid of restriction enzymes underlines the importance of creating robust informatics tools that can not only identify *existing *restriction enzyme recognition sites but also support the *design *of new restriction sites at a desired location without changing the protein sequence through the use of silent mutations. Based on this rationale, we designed a novel fast pattern finding algorithm, named Inverse Codon Replacement Pattern finder (ICRPfinder). In this paper, we describe several key aspects of the ICRPfinder algorithm and provide sample results using ICRPfinder to find or create restriction enzyme recognition sites in target coding sequences. ICRPfinder is a web-based application and can be accessed using a standard internet browser or run as a stand alone application on a local machine.

## Implementation

### Algorithm

#### Challenges of brute force approach

**Figure 1:**
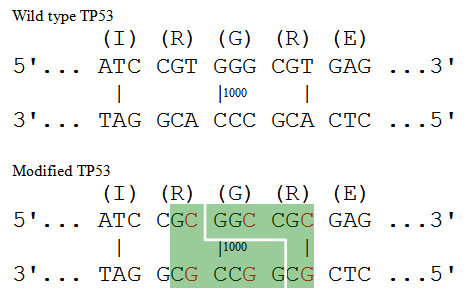
**Create a DNA pattern using silent mutations**. Wild type TP53 and modified TP53 with a restriction enzyme NotI recognition site (GCGGCCGC) in the green rectangle. Base pairs with red colors are silent mutations. Number 1000 represent the location of the base pair in TP53 DNA sequence.

To design novel restriction enzyme recognition sites, a brute force approach would start with the translated protein sequence. It would find all codons that encode each amino acid in the protein sequence; it would generate an exhaustive list of ordered codon combinations; then, it would look for the desired pattern (a short DNA sequence) in each candidate DNA sequence. A challenge of this brute force approach is that the number of DNA sequences which can be translated into the target protein grows exponentially with the length of the protein. For example, assume a researcher wants to find a recognition site for the restriction enzyme NotI, GCGGCCGC, in the human TP53 protein (Figure 1). TP53 has 393 amino acids and it does not include the desired sequence, GCGGCCGC. Consider a 5 amino acid subsequence, IRGRE, located at the position 332-336 of the TP53 protein. There are 3 codons (ATT, ATC, and ATA) which can be translated into the amino acid Ile/I, 6 codons for Arg/R, 4 codons for Gly/G, 6 codons for Arg/R, and 2 codons for Glu/E (Table 1). A total of 864 (3 × 6 × 4 × 6 × 2 = 864) possible DNA sequences can be constructed encoding for this 5-amino acid sequence alone. As the average number of codons coding for each amino acid is close to 3 (64/20≈3), given a protein of length *n*, the number of possible DNA sequences is approximately 3^n^. Since the computational complexity is exponential (O(n*3^n^)), the brute force look-up approach becomes intractable as the length *n *of a protein increases.

**Table 1:** Possible DNA sequences required to be looked-up in the brute force approach

**Possible codons at each Position**				
**1**	**2**	**3**	**4**	**5**

I	R	G	R	E

ATT	CGT	GGT	CGT	GAA

ATC	CGC	GGC	CGC	GAG

ATA	CGA	GGA	CGA	

	CGG	GGG	CGG	

	AGA		AGA	

	AGG		AGG	

There are total 864 (3 × 6 × 4 × 6 × 2 = 864) possible DNA sequences created by combining codons from each position. Any combination, for example ATTCGTGGTCGTGAA, or the original sequence, ATCCGTGGGCGTGAG, can be translated to the same amino acid sequence IRGRE. The brute force approach is not practical because of the exponential growth in the number of possible sequences.

#### Proposed non-brute force approaches

We propose two approaches that avoid the generation of all possible DNA sequences as required by the brute force approach. For illustration, we continue using the example with NotI, with the GCGGCCGC restriction site pattern.

##### Approach 1

At each position (amino acid) of the target protein all possible codons are compared to the NotI sequence. If no codon matches then we proceed to the next position and start a new match. If one codon is matched, however, then we continue to check the neighboring amino acids for potential complete matches. As numerous locally unmatched DNA sequences are discarded, the computational complexity is decreased to O(n*3n).

##### Approach 2

An alternative approach would be to "translate" the NotI sequence into three amino acid sequences, one for each reading frame. For example, we shift and "translate" the NotI sequence as:

GCG GCC GC -> "AA" with "GC" right overhang;

G CGG CCG C -> "RP" with "G" left overhang and "C" right overhang;

GC GGC CGC -> "GR" with "GC" left overhang.

There might be "overhangs" of one or two nucleotides at the right, left or both sides. We compare the "translated" NotI sequence to the target protein sequence first. If the "translated" sequence and the target protein sequence are matched, continue to compare the left and right overhangs.

We compare the "AA", "RP" and "GR" one by one to the target "IRGRE", and find "GR" in the target sequence ("IR *GR *E"). Then we proceed to investigate whether the left overhang "GC" is also matched by the left amino acid "R" ("I *R *GRE"). There are six codons for the amino acid R, and one of them, CGC, matches the left overhang "GC" (C *GC*). This means that either the NotI recognition sequence (GCGGCCGC) is found in the original target protein TP53 or that one can create a recognition site in the target protein by using a few silent mutations. That is the solution. In this approach the computational complexity is O(n*3), so execution time falls dramatically. We chose Approach 2, which is more efficient, for implementation. Since we "translate" the object DNA pattern into an amino acid sequence and compare it to the target protein sequence, we call this algorithm Inverse Codon Replacement Pattern finder (ICRPfinder). The object pattern can be a restriction enzyme recognition sequence, or any other nucleic acid sequences of interest, such as promoter sequences.

### Source code

ICRPfinder is an open source JavaScript application. It runs on most commonly used Web browsers, including Netscape Navigator, Internet Explorer, Firefox and Safari. ICRPfinder can be easily mirrored on a user's Web site, or run on a local computer off-line. The code is freely available under the GNU General Public License.

ICRPfinder includes 2406 common restriction site patterns, and additional sequence patterns can be input by the user. All information regarding the restriction enzymes used in ICRPfinder was retrieved from REBASE [16] version 906.

## Results and Discussion

**Figure 2:**
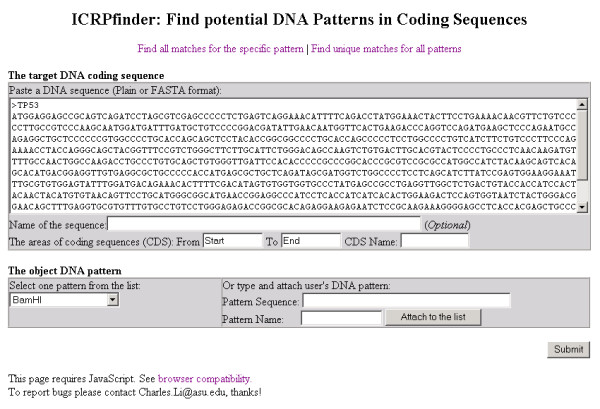
**The GUI of ICRPfinder for specific pattern analysis**. A plain or FASTA format target DNA sequence can be accepted. Restriction enzymes can be chosen, and also users' pattern sequences can be accepted.

Find all possible potential matches for a specific pattern Figure 2 illustrates the Graphical User Interface (GUI) of the Web application in which users input the target DNA coding sequence and object pattern and configure the parameters. From the menu bar, users can select from two options: (1) find all potential matches for a specific pattern; or (2) find unique matches for all patterns. Below the menu bar, a text box allows for entering and configuring the target DNA coding sequence. Nucleotide sequences in plain text or FASTA format are accepted. The coding sequences (CDS) can be specified and accepted one by one if the input sequence includes not only CDS but also introns or sequences from bacterial vectors. Below the target DNA box, another text box allows the configuration of the object DNA pattern. A list of restriction enzyme recognition sites is pre-loaded as default patterns. User-defined patterns as well as their names can also be accepted.

**Figure 3:**
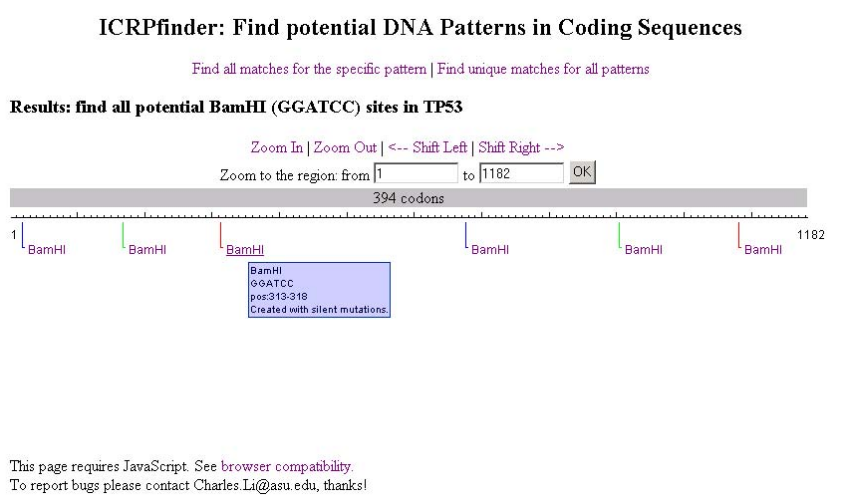
**The results of finding possible recognition sites for a specific restriction enzyme**. BamHI recognition sites are displayed. Lines with different colors are employed for better visualization, especially when results are close to each other. The information for the sequence and position for each recognition site will popup when the mouse hovers over the sequence.

Figure 3 illustrates the functionality for finding or creating all possible recognition sites in the target coding sequence for the specific pattern. The results of all possible recognition sites for the specific restriction enzyme BamHI (GGATCC) in the TP53 DNA sequence are displayed. In this example, the recognition sites are created with the silent mutations strategy. As discussed previously, the existing tools such as NEBcutter or WatCut do not support this functionality, whereas ICRPfinder can create 6 BamHI recognition sites without changing the protein sequence encoded. The detailed information is displayed in a tooltip by moving the mouse over the recognition sites.

The results are rendered graphically, and appear in text format if the target coding sequence is short or by zooming in on the results.

### Create unique patterns around a specific position

**Figure 4:**
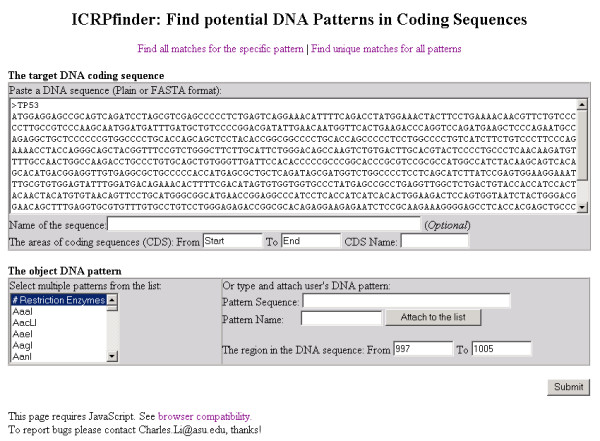
**The GUI of ICRPfinder for specific position analysis**. The specific position can be accepted. All patterns, including restriction enzymes and user's patterns are analyzed.

Figure 4 illustrates the GUI by which users can analyze a DNA sequence around a specific position. There are two main differences between this and Figure 2. The first is that only the sequence within a short range of the specified position is displayed. The range is expected to be no more than 20 nucleotides since recognition sites are normally only several nucleotides in length, but the range is not limited to that size so as not to restrict the possible use of this tool for other purposes. The second difference is that patterns do not need to be selected, since all patterns are analyzed. This function is useful to find all patterns which are *located only around a specific position *and nowhere else in the target coding sequence. As only one possible matched site is found or created, the other parts of the target coding sequence will be unaffected.

**Figure 5:**
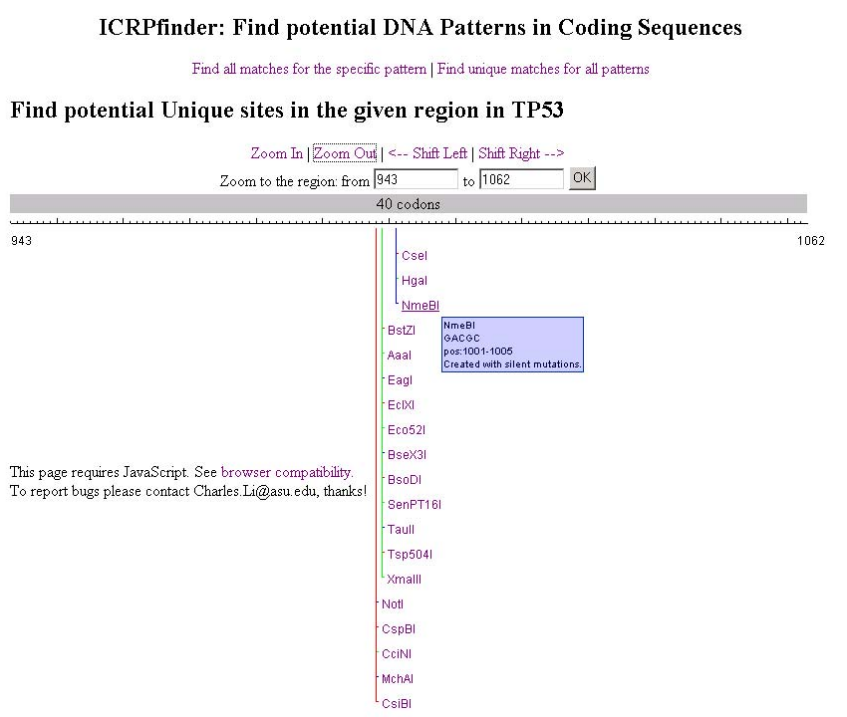
**The results of creating unique recognition sites around a specific position**. All enzymes which recognise only one site in the target TP53 DNA sequence are displayed. Lines with different colors are utilized for better visualization, especially when results are close to each other. The information for the sequence and position for each recognition site will popup when the mouse hovers over the sequence.

Figure 5 illustrates the results for unique recognition sites around a specific position in the target TP53 DNA sequence. In the range of positions 997-1005 (9 nucleotide length) ICRPfinder creates 3 potential recognition sites and at each site the related restriction enzyme cleaves the TP53 gene only around the specific position.

### Performance benchmarking

A benchmark test to evaluate the relative performance of ICRPfinder, NEBCutter and WatCut was performed. The experiments were performed on a server with 1.86 GHz of CPU and 4 GB of RAM. The server has excellent network connectivity with a speed of 1.0 Gbps on the .edu domain. The data used in the experiments are the TP53 DNA coding sequence and a random coding sequence created by SMS [9]. The lengths of the two sequences are 1182 nt and 12000 nt respectively. For WatCut, only 100 bp were used, since WatCut limits the length of the input coding sequence to 100 bp. The details of searching and response times are shown in table 2.

**Table 2:** Performance benchmark for ICRPfinder, NEBCutter and WatCut

	**Details of searching**			**Response time (sec)**	
	**mode**	**patterns**	**region**	**TP53 CDS (1182 bp)**	**Random CDS (12,000 bp)**

ICRPfinder 1	Wild type, Silent mutation	1	Whole coding sequence	0.03	0.21

ICRPfinder 2	Wild type, Silent mutation	2406	Specific region	0.53	0.52

NEBCutter	Wild type	1	Whole coding sequence	3-6	6-12

WatCut	Wild type, Silent mutation	246 (generic enzymes)	Whole coding sequence	2-3*	

The performance of ICRPfinder, NEBCutter and WatCut are compared on same data. In the first column ICRPfinder 1 stands for the function to find all potential positions for a specific pattern and ICRPfinder 2 stands for the function to find all unique patterns in a given region.

*Only 100 bp were used to run WatCut, since the application limits the length of the input coding sequence to 100 bp.

Typical response times for ICRPfinder were under 1 second, while for NEBCutter the repose times were between 6 and 12 seconds. There could be two reasons why response times of NEBCutter are quite long compared to ICRPfinder. The first reason is the delay due to network transport. Although the server has excellent network connectivity, the transport latency can not be neglected. The second reason could be due to GUI rendering of results. The GUI rendering in NEBCutter is relatively slow as NEBCutter has to choose and display partial results according to the width of the display region. The NEBCutter response time also can vary depending on the number of results to be displayed. It appears that in the zoomed out display, NEBCutter only displays a fraction of the possible restriction enzyme cut sites, while ICRPfinder displays all the restriction sites. If NEBCutter were to display all the restriction sites, we can presume that the response time for NEBCutter would be even slower.

For WatCut, the response times were 2-3 seconds; however, one must note that only a small fraction of the sequence was used, since WatCut limits the input coding sequence to 100 bp. WatCut was run with the default set of enzymes (246), while ICRPfinder was run with a larger default set, of 2406 enzymes.

## Conclusion

ICRPfinder is a powerful tool for finding or creating a specific DNA sequence in a given coding sequence. It translates both the target coding sequence and object DNA pattern to amino acid sequences and finds the matches between two amino acid sequences. In addition to finding restriction enzyme recognition sites in the given target coding sequence, ICRPfinder can create recognition sites without changing the translated protein sequence. In addition to restriction enzyme recognition sites, as a general purposed tool, ICRPfinder can also accept users' DNA patterns and then help to analyze any DNA-protein binding events by given DNA patterns. The non-brute force approach with DNA translating can dramatically decrease the computation time for the pattern finding/creating process. Since ICRPfinder is a browser-based application, it can run on any platform, in on-line or off-line mode. This tool should be useful for experimental biologists who manipulate or synthesize large DNA sequences or even whole genomes.

## Availability and requirements

Project name: ICRPfinder

Project home page: 

Operating system(s): Platform independent

Programming language: JavaScript and DHTML

License: GNU GPL

Any restrictions to use by non-academics: See GNU GPL license for details

## Authors' contributions

CL and XZ conceived this work, CL wrote the manuscript, YL performed the test, VD advised this work and revised the manuscript, and PS gave advice and revised the manuscript. All authors read and approved the final manuscript.
